# Systematic profiling identifies *PDLIM2* as a novel prognostic predictor for oesophageal squamous cell carcinoma (ESCC)

**DOI:** 10.1111/jcmm.14491

**Published:** 2019-06-20

**Authors:** Guiqin Song, Jun Xu, Lang He, Xiao Sun, Rong Xiong, Yuxi Luo, Xin Hu, Ruolan Zhang, Qiuju Yue, Kang Liu, Gang Feng

**Affiliations:** ^1^ Institute of Tissue Engineering and Stem Cells Nanchong Central Hospital, The Second Clinical Medical College, North Sichuan Medical College Nanchong China; ^2^ Department of Biology North Sichuan Medical College Nanchong China; ^3^ Department of Thoracic Surgery Nanchong Central Hospital, The Second Clinical Medical College, North Sichuan Medical College Nanchong China; ^4^ Department of Oncology The Fifth People's Hospital of Chengdu, The Second Clinical Medical School, Chengdu University of Traditional Chinese Medicine Chengdu China; ^5^ Precision Medicine Center Nanchong Central Hospital Nanchong China; ^6^ The First Clinical College of Anhui Medical University Hefei China

**Keywords:** copy number alteration, methylation, oesophageal squamous cell carcinoma, *PDLIM2*, prognosis

## Abstract

Till now, no appropriate biomarkers for high‐risk population screening and prognosis prediction have been identified for patients with oesophageal squamous cell carcinoma (ESCC). In this study, by the combined use of data from the Gene Expression Omnibus (GEO) datasets and The Cancer Genome Atlas (TCGA)‐oesophageal carcinoma (ESCA), we aimed to screen dysregulated genes with prognostic value in ESCC and the genetic and epigenetic alterations underlying the dysregulation. About 222 genes that had at least fourfold change in ESCC compared with adjacent normal tissues were identified using the microarray data in GDS3838. Among these genes, only *PDLIM2* was associated with nodal invasion and overall survival (OS) at the same time. The high *PDLIM2* expression group had significantly longer OS and its expression was independently associated with better OS (HR: 0.64, 95% CI: 0.43‐0.95, *P* = 0.03), after adjustment for gender and pathologic stages. The expression of its exon 7/8/9/10 had the highest AUC value (0.724) and better prognostic value (HR: 0.43, 95% CI: 0.22‐0.83, *P* = 0.01) than total *PDLIM2* expression. *PDLIM2* DNA copy deletion was common in ESCC and was associated with decreased gene expression. The methylation status of two CpG sites (cg23696886 and cg20449614) in the proximal promoter region of *PDLIM2* showed a moderate negative correlation with the gene expression in *PDLIM2* copy neutral/amplification group. In conclusion, we infer that *PDLIM2* expression might be a novel prognostic indicator for ESCC patients. Its exon 7/8/9/10 expression had the best prognostic value. Its down‐regulation might be associated with gene‐level copy deletion and promoter hypermethylation.

## 1 INTRODUCTION

Oesophageal squamous cell carcinoma (ESCC) is the dominant histological subtype of oesophageal carcinoma (ESCA) and is a highly aggressive malignancy because of the strong potential of invasion and metastasis.^1^ Therefore, the overall 5‐year survival rate after surgery and chemotherapy is still low, ranging from 15% to 25%.^1^, ^2^ This disease has remarkable geographic distribution, showing particularly high incidence and mortality rates in China and some other Asian countries.^2^ In the past decades, a series of studies have been performed to understand the molecular mechanisms underlying the pathological development of ESCC.^3^, ^4^, ^5^ However, no appropriate biomarkers for high‐risk population screening and prognosis prediction have been identified. TNM stage system is still the most reliable tool to stratify patients for making treatment plan and to predict prognosis. In clinical practice, ESCC patients with the same TNM stage and receiving the same therapy might have significantly different survival outcome.^6^, ^7^, ^8^ To improve prognosis, it is imperative to look for reliable biomarkers for identifying high‐risk patients who require intensive follow‐up and therapeutic support.

Over the last decade, The Cancer Genome Atlas (TCGA) project provided comprehensive clinical, genetic, epigenetic and pathological data to illuminate the landscapes of 33 types of primary tumours based on cancerous and normal tissues from over 10,000 patients.^9^, ^10^ TCGA‐ESCA is a part of the TCGA project that contains data from around 200 ESCA (100 adenocarcinomas and 100 ESCC respectively) patients, which enables us to explore the association between cancer phenotypes and genotypes. The long‐term survival data also provided a reliable platform to explore prognostic biomarkers.^11^ However, the number of normal samples in this dataset is relatively small (N = 10 for adenocarcinoma and N = 3 for ESCC), which makes it difficult to analyse the dysregulated genes (DEGs). The Gene Expression Omnibus (GEO) DataSets (https://www.ncbi.nlm.nih.gov/geo/info/datasets.html) store a large number of gene expression data from original submitter‐supplied series, samples and platforms, thus providing an optimal data reservoir to explore gene expression profiles.

In this study, by the combined use of data from GEO datasets and TCGA‐ESCA, we aimed to screen the significantly dysregulated genes and to explore their prognostic value in ESCC. In addition, the potential genetic and epigenetic alterations associated with the candidate prognostic markers were also investigated.

## 2 MATERIALS AND METHODS

### 2.1 Data mining in GEO datasets

The normalized data of one previous Affymetrix HG‐U133A 2.0 gene expression array that compared gene expression in 17 micro‐dissected ESCC tumours and matched adjacent normal tissue pairs were downloaded from: (https://www.ncbi.nlm.nih.gov/sites/GDSbrowser?acc=GDS3838).^12^ The data were loaded into NetworkAnalyst for re‐analysis, which is a visual analytics platform for comprehensive gene expression profiling.^13^ Only the genes had log2 fold change ≥2 and adjusted *P* < 0.05 were identified as the candidate DEGs.

### 2.2 Data mining in TCGA‐ESCA

The level‐3 data in TCGA‐ESCA were acquired with the use of the UCSC Xena browser (https://xenabrowser.net/).^14^ Only the ESCC subset (TCGA‐ESCC) that with RNA‐seq data of gene expression was extracted and used in this study. None of the patients in this subset received neoadjuvant treatment. After screening with these criteria, 95 cases of primary ESCC were identified. The clinicopathological data, including age at initial diagnosis, gender, histological grade, the history of oesophageal cancer, pathological nodal (pN) status, pathological stages, reflux history, smoking history, post‐operative drug therapy, radiation therapy, alcohol consumption per week (defined as frequency of alcohol consumption/week * amount of alcohol consumption/day) and OS survival data were downloaded for re‐analysis.

The genomic data of the ESCC cases, including the RNA‐seq data of total gene expression, the RNA‐seq data of exon expression of a specific gene, the methylation status, DNA copy number alterations (CNAs) and somatic mutations of targeting genes were also extracted. Briefly, the methylation status of target genes was examined using Infinium HumanMethylation450 BeadChip, which covers over 450,000 methylation sites across the genome per sample. Beta values were calculated to determine the methylation level, with the formula: *β* = M/(U+M+100). In the equation, M and U refer to the fluorescence level of the methylation probe and unmethylated probe respectively. Genomic CNAs were calculated by an algorithm called gene‐level thresholded Genomic Identification of Significant Targets in Cancer 2.0 (GISTIC2), which aims to assign discrete numbers to the gene fragments by thresholding the data.^15^ The alterations in a gene were generally classified into five groups, including copy neutral (0), low‐level copy gain (+1), high‐level amplification (+2), heterozygous loss (−1) or homozygous deletion (−2). Somatic mutations include both single‐nucleotide polymorphisms and (SNPs) small insertions and deletions (INDELs).

### 2.3 In silico analysis of the alternative transcription of PDLIM2

The alternative transcripts of *PDLIM2* in oesophageal cancer were analysed using ISOexpresso (http://wiki.tgilab.org/ISOexpresso/), which is a web‐based platform for isoform‐level expression analysis among samples in TCGA.^16^ The genes were matched with HGNC gene, Ensembl release 74 and UCSC refGene, while the isoform information of the genes is collected from UniProt ID mapping data and UCSC knownGene tables 16. The relative expression level of each transcript was quantified using transcripts per kilobase million.

### 2.4 Statistical analysis

Data integration and analysis were conducted using spss 25.0 software package (SPSS Inc, Chicago, IL), together with GraphPad Prism 7.04 (GraphPad Inc, La Jolla, CA). The difference in gene expression between groups was compared using unpair *t* test with Welch's correction. Receiver operating characteristic (ROC) curve was generated and area under the curve (AUC) was calculated to assess the predictive value of *PDLIM2* and its exon expression for OS. Log‐rank test was performed to examine the significance of the difference between the Kaplan‐Meier OS curves. Stepwise regression was conducted to compare the predictive value of total *PDLIM2* expression and its exon 7/8/9/10 expression in terms of OS. The prognostic value of *PDLIM2* and its exon expression were assessed using the univariate and multivariate Cox regression models. Linear regression analysis was performed and the Pearson's correlation coefficient was calculated to assess the correlation between total *PDLIM2* expression and the expression of its exons or the methylation status of the CpG sites in its DNA locus. *P* < 0.05 was considered statistically significant.

## 3 RESULTS

### 3.1 Screening of significantly dysregulated genes in ESCC

**Figure 1:**
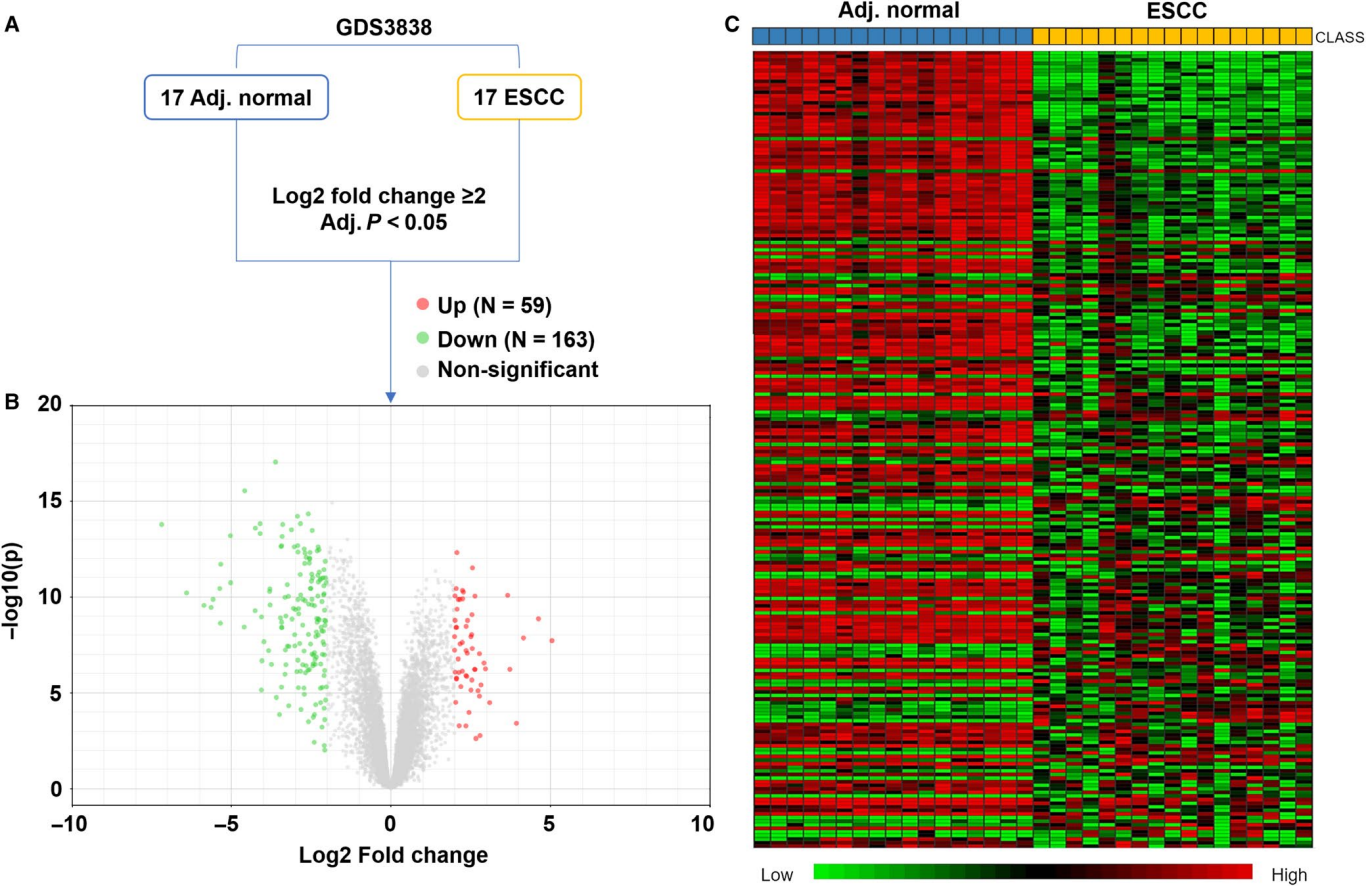
Screening of significantly dysregulated genes in oesophageal squamous cell carcinoma (ESCC). (A), The screening criteria and process to identify the dysregulated genes (DEGs) in GDS3838. (B), Volcano plot chart showing the distribution of the DEGs. Up‐regulated genes are RED; down‐regulated genes are GREEN. (C), Heatmap of the DEGs between the 17 ESCC tumour tissues and matched normal tissues

Using the microarray data from GDS3838, we screened the significant DEGs between the 17 cases of ESCC tumour tissues and matched normal tissues, with the following criteria: log2 fold change ≥2 and adjusted *P* < 0.05 (Figure 1A). Results showed that there were 59 up‐regulated and 163 down‐regulated genes in ESCC compared to adjacent normal tissues (Figure 1B,C). The details of the 222 DEGs were provided in Table S1.

### 3.2 Screening of OS‐related DEGs in ESCC

**Figure 2:**
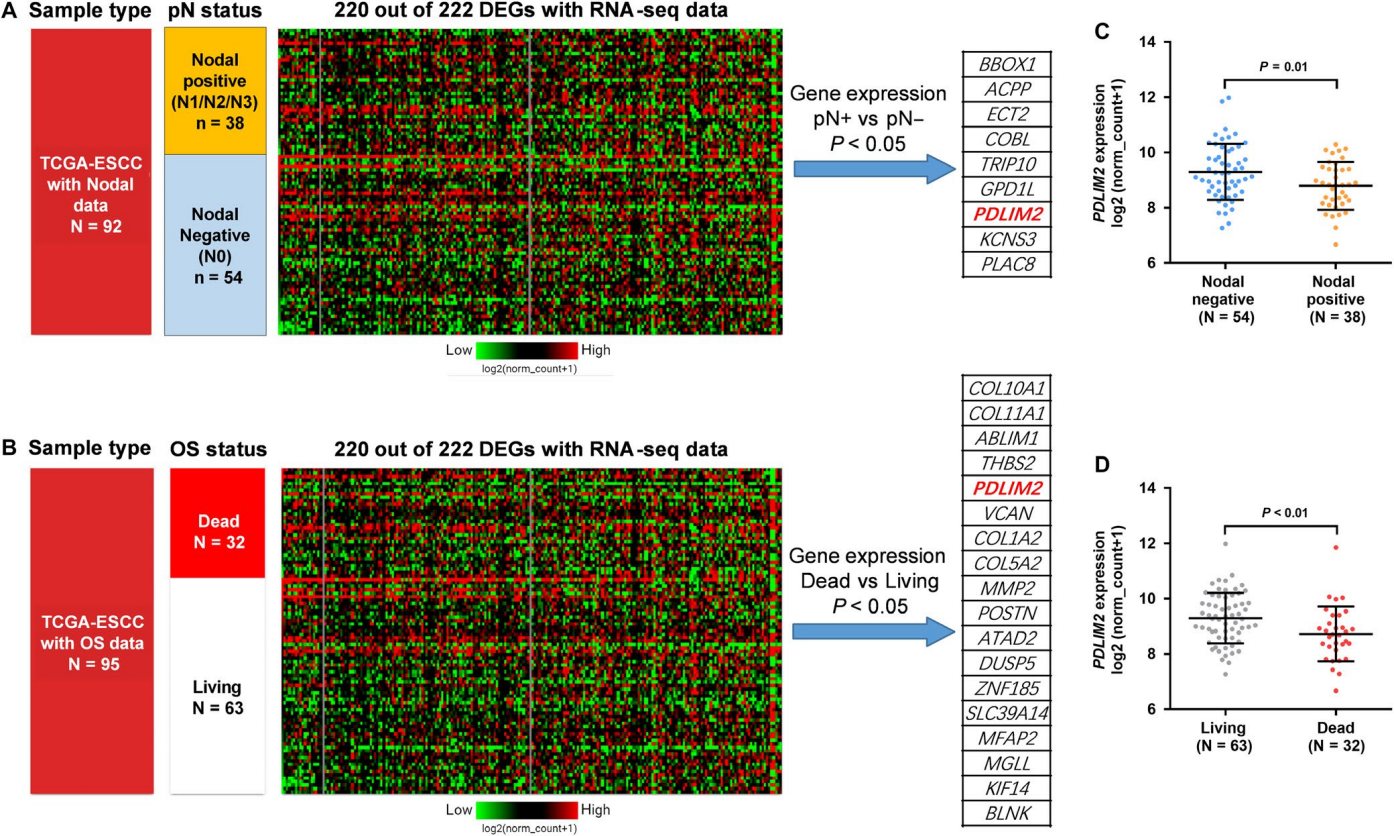
Screening of overall survival (OS) related dysregulated genes (DEGs) in oesophageal squamous cell carcinoma (ESCC). (A,B), Heatmap showing the expression profile of 220 DEGs with RNA‐seq data in TCGA‐ESCC between nodal positive and nodal negative cases (A) and between the living and dead cases (B). *P* value was calculated by performing unpair *t *test with Welch's correction. (C,D), Plot charts comparing the expression of *PDLIM2* between nodal positive and nodal negative cases (C) and between the living and dead cases (D)

In the ESCC subset of TCGA‐ESCA, we found that 220 of the 222 DEGs had RNA‐seq data of gene expression. Lymph nodal invasion is a critical hallmark of the progression of ESCC and direct increases the risk of unfavourable survival.^17^ Then, we examined the expression profiles of the 220 DEGs in ESCC patients grouped according to their nodal invasion status (Figure 2A) and OS status (Figure 2B). There were nine genes that were significantly dysregulated in nodal positive cases, including *BBOX1, ACPP, ECT2, COBL, TRIP10, GPD1L, PDLIM2, KCNS3* and *PLAC8* (Figure 2A), while the dysregulation of 18 genes (*COL10A1, COL11A1, ABLIM1, THBS2, PDLIM2, VCAN, COL1A2, COL5A2, MMP2, POSTN, ATAD2, DUSP5, ZNF185, SLC39A14, MFAP2, MGLL, KIF14* and *BLNK*) were associated with OS status (Figure 2B). Interestingly, among these genes, only *PDLIM2* was associated with nodal invasion and survival outcome at the same time. The nodal positive cases and dead cases had significantly down‐regulated *PDLIM2* expression compared to the respective control groups (*P* = 0.01 and *P* < 0.01 respectively) (Figure 2C,D).

We also generated ROC curves and calculated the AUCs of the 18 OS‐related genes in Figure 2B, by setting living as the status variable. The AUCs of *PDLIM2* (0.684) ranked third among the genes, while *ATAD2* and *COL10A1* showed higher AUCs (0.692 and 0.691 respectively) (Figure S1). However, we then checked their expression profile and found that these two genes were significantly up‐regulated in ESCC compared with adjacent normal tissues (Table S1) and thus were abandoned for analysis.

The clinicopathological and survival data extracted from TCGA‐ESCC were provided in Table S2. Ninety‐five ESCC patients were divided into two groups according to median *PDLIM2* expression. Their clinicopathological parameters were compared in Table 1. Results showed that these two groups had similar clinicopathological profiles, expect the ratio of survival. The high *PDLIM2* expression had a significantly lower ratio of death (9/48 vs 23/47, *P* < 0.01) (Table 1).

**Table 1:** Comparison of the clinicopathological parameters between oesophageal squamous cell carcinoma patients with high and low *PDLIM2* expression

Parameters		*PDLIM2* expression		*P* value
		High (N = 48)	Low (N = 47)	
Age (mean ± SEM)		58.63 ± 1.57	58.02 ± 1.42	0.78
Gender	Female	5	9	0.26
	Male	43	38	
Histological grade	G1/G2	35	29	0.45
	G3	9	12	
	GX	4	6	
Pathological stage	I/II	34	28	0.28
	III/IV	13	18	
	null	1	1	
Pathological N	N0	31	23	0.14
	N1+	15	23	
	NX/null	2	1	
Reflux history	No	32	31	0.77
	Yes	8	6	
	Null	8	10	
Smoking history	1	14	18	0.28
	2‐4	33	26	
	Null	1	3	
Radiation therapy	No	25	30	0.28
	Yes	20	14	
	Null	3	3	
Post‐operative drug therapy	No	32	34	0.64
	Yes	14	11	
	Null	2	2	
Recurrence after primary therapy	Otherwise	28	30	0.33
	New tumor	14	9	
	Null	6	8	
Living status	Living	39	24	<0.01
	Dead	9	23	

Note

Smoking history: 1: lifelong non‐smoker; 2: current smoker; 3. Current reformed smoker (for >15 y); 4. Current reformed smoker (for ≤15 y); N1+: N1/N2/N3; NX: Regional lymph nodes cannot be assessed; null: no data recorded.

**Figure 3:**
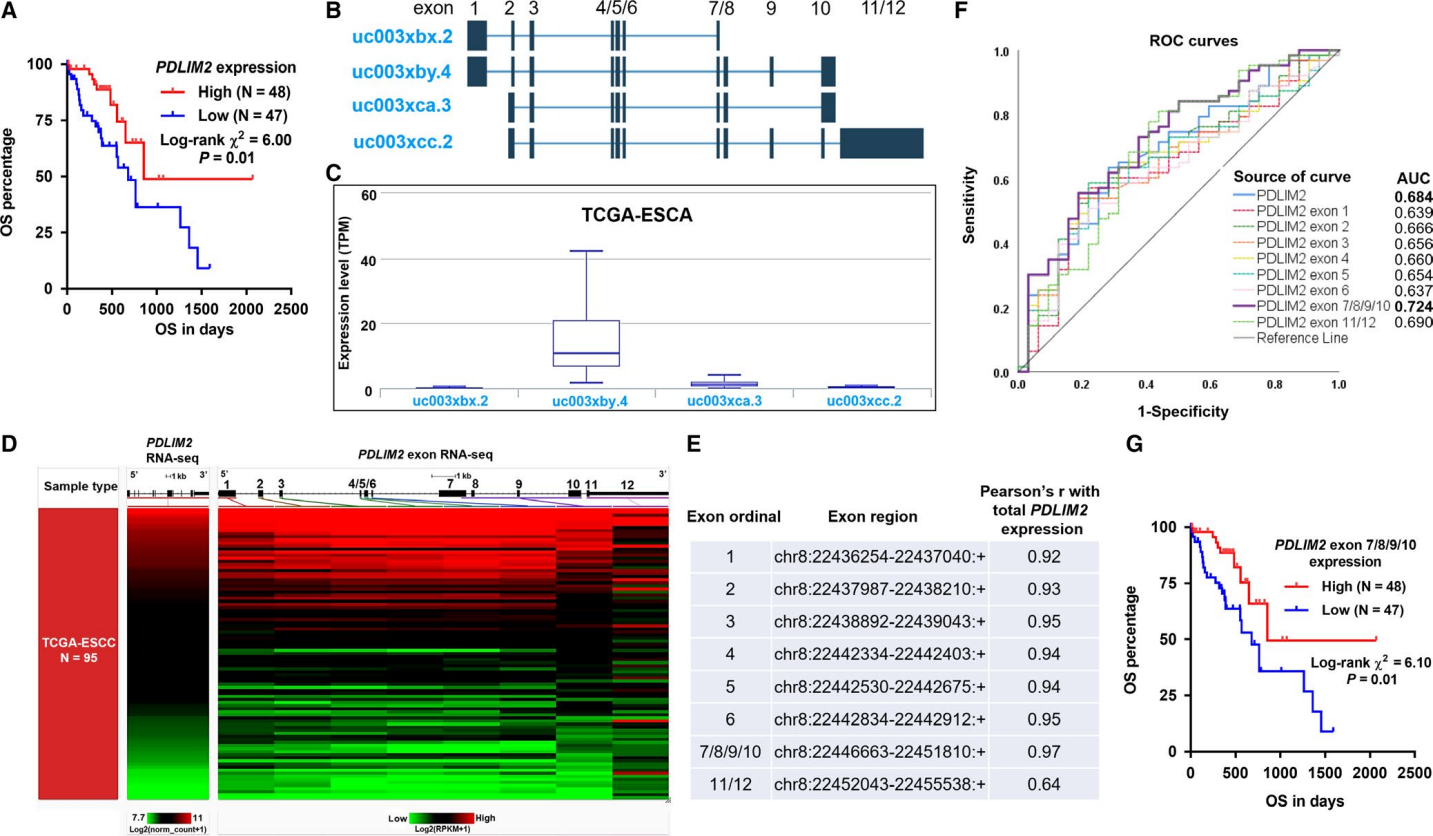
The expression of *PDLIM2* exon 7/8/9/10 might be a better prognostic marker than total *PDLIM2* expression in terms of overall survival (OS). (A), Kaplan‐Meier curves of OS of oesophageal squamous cell carcinoma (ESCC) patients. The patients were separated into two groups according to median *PDLIM2* expression. (B), Representative exon structure of the major *PDLIM2* transcripts (uc003xbx.2, uc003xby.4, uc003xca.3 and uc003xcc.2) in TCGA‐ESCA. (C), The expression profile of the four major *PDLIM2* transcripts in TCGA‐ESCA. (D), Heatmap showing the correlation between *PDLIM2* expression in total and the expression of its exons. (E), Summary of the Pearson's correlation coefficient between *PDLIM2* expression in total and the expression of its exons. (F), Receiver operating characteristic (ROC) analysis of the predictive value of different *PDLIM2* exon expression and total *PDLIM2* expression in terms of OS. (G), Kaplan‐Meier curves of OS of ESCC patients. The patients were separated into two groups according to median *PDLIM2* exon 7/8/9/10 expression

By generating Kaplan‐Meier survival curves according to the median expression of *PDLIM2*, we found that the high *PDLIM2* expression group had significantly longer OS (*P* = 0.01) (Figure 3A). Univariate analysis showed that male patients, advanced pathological stages and decreased *PDLIM2* expression were the risk factors of unfavourable survival (Table 2). The following multivariate analysis confirmed that total *PDLIM2* expression was independently associated with better OS in ESCC patients (HR: 0.64, 95% CI: 0.43‐0.95, *P* = 0.03) (Table 2), after the adjustment for gender and pathologic stages.

**Table 2:** Univariate and multivariate analysis of the prognostic value of *PDLIM2* in terms of overall survival in oesophageal squamous cell carcinoma patients

Parameters	Univariate analysis				Multivariate analysis			
	*P*	HR	95% CI (lower/upper)		*P*	HR	95% CI (lower/upper)	
Age	0.05	1.04	1.00	1.07				
Gender								
Female vs Male	**0.03**	0.19	0.04	0.82				
Histologic grade								
G1/G2 vs G3	0.45	1.45	0.55	3.85				
History of oesophageal cancer								
No vs Yes	0.10	0.36	0.11	1.21				
Pathologic stages								
I/II vs III/IV	**0.02**	0.42	0.20	0.86				
Reflux History								
No vs Yes	0.39	0.68	0.29	1.63				
Smoking History								
Yes vs No	0.32	1.54	0.66	3.59				
Radiation therapy								
No vs Yes	0.67	0.83	0.36	1.95				
Post‐operative drug therapy								
No vs Yes	0.56	1.31	0.53	3.25				
Alcohol consumption/wk	0.41	1.01	0.99	1.02				
Total *PDLIM2* expression	**0.04**	0.69	0.48	0.99	**0.03**	0.64	0.43	0.95
*PDLIM2* exon 7/8/9/10 expression	**0.02**	0.48	0.26	0.87	**0.01**	0.43	0.22	0.83

Note

Multivariate analysis was performed by setting gender, pathological stages and total *PDLIM2* expression or gender, pathological stages and *PDLIM2* exon 7/8/9/10 expression as covariates respectively.

Bold indicates *P* < 0.05.

### 3.3 The expression of PDLIM2 exon 7/8/9/10 showed better prognostic value than total PDLIM2 expression

The accuracy of a prognostic marker can be generally divided into five categories on the basis of the AUC values 0.50‐0.60 = fail，0.60‐0.70 = poor, 0.70‐0.80 = fair, 0.80‐0.90 = good and 0.90‐1 = excellent.^18^ Although we found that *PDLIM2* expression might have potential prognostic value in terms of OS, ROC analysis showed that its expression only had an AUC value of 0.684 (Figure S1), which suggests a poor accuracy of a prognostic test. *PDLIM2* gene contains 12 exons and spans about 20 kb in human genome.^19^ Besides, alternative splicing and subsequent transcript variants encoding multiple isoforms have been observed for this gene.^19^ Therefore, the prognostic value of total *PDLIM2* expression might be impaired by the variation of its transcripts. By checking alternative splicing events in TCGA‐ESCA, we found that among the four major transcripts of *PDLIM2* (uc003xbx.2, uc003xby.4, uc003xca.3 and uc003xcc.2) (Figure 3B), the canonical uc003xby.4 isoform, which contains exon 1‐10 is the dominant transcript in TCGA‐ESCA (Figure 3C). Then, we checked the expression profile of *PDLIM2* exons and analysed their correlation with total *PDLIM2* expression (Figure 3D,E). Data in TCGA showed that among the 12 exons of *PDLIM2*, exon 7/8/9/10 and exon 11/12 tend to be transcribed together and their expression data were provided as a whole. Therefore, the expression of these two segments was treated as two expression units. Correlation analysis showed that except chr8:22452043‐22455538:+, which represent the expression of exon 11/12, all other exons were very strongly correlated with total *PDLIM2* expression (Pearson's *r* > 0.9, Figure 3D,E). The exon region, chr8:22446663‐22451810:+, which represents the expression of exon 7/8/9/10 had the strongest correlation with total *PDLIM2* expression (Pearson's *r* = 0.97; Figure 3E). ROC analysis showed that the expression of exon 7/8/9/10 had the highest AUC value (0.724) (Figure 3F, bold purple), which suggests that it might be a fair prognostic marker. Compared with the AUC value of total *PDLIM2* expression (0.684), this region might be a better marker. To test this hypothesis, we further performed a stepwise regression analysis by setting OS as the dependent variable. Results showed that the expression of exon 7/8/9/10 is a better predictor in the model than total *PDLIM2* expression in predicting the OS of ESCC patients (Table S3). Multivariate analysis further confirmed the independent prognostic value of exon 7/8/9/10 expression in terms of OS (HR: 0.43, 95% CI: 0.22‐0.83, *P* = 0.01) (Table 2).

### 3.4 In silico analysis of the potential genetic and epigenetic alterations associated with PDLIM2 dysregulation

**Figure 4:**
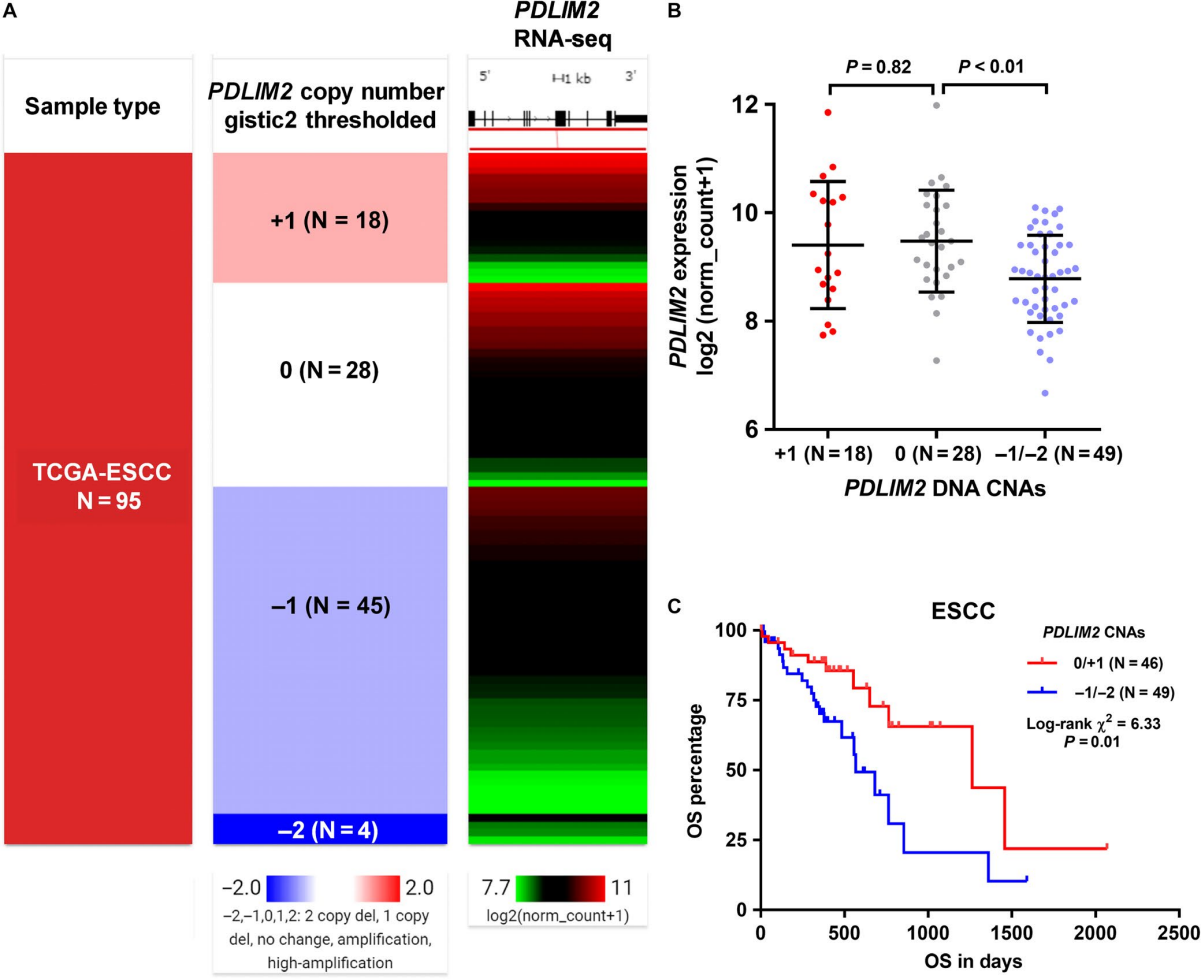
*PDLIM2* DNA CNAs, its expression and patients’ survival. (A), Heatmap showing the correlation between *PDLIM2* DNA CNAs and its expression. (B), Plot chart showing the expression of *PDLIM2* in different CNA groups. CNAs were defined as: copy‐neutral (0), low‐level copy gain (+1), high‐level amplification (+2), heterozygous loss (−1) or homozygous deletion (−2). (C), Kaplan‐Meier curves of OS of ESCC patients. The patients were separated into two groups according to CNA status

In TCGA‐ESCC, all 95 ESCC cases included in this study had *PDLIM2* CNA data available. Data showed that *PDLIM2* CNAs were quite common. Among the 95 ESCC cases, 18 cases (18.9%) had *PDLIM2* amplification (+1), 45 cases (47.4%) had heterozygous deletion (−1) and 4 cases (4.2%) had homozygous deletion (−2). Only 28 cases (29.5%) were copy neutral (Figure 4A). Then, we examined the correlation between *PDLIM2* expression and its DNA CNAs. *PDLIM2* amplification did not necessarily result in up‐regulated transcription, compared to copy neutral cases (*P* = 0.82, Figure 4B). In comparison, its DNA deletion was associated with significantly lower gene expression, compared with the copy neutral cases (*P* < 0.01, Figure 4B). Kaplan‐Meier survival curves showed that the group with *PDLIM2* DNA deletion had significantly inferior OS, compared to the amplification/copy neutral group (*P* < 0.01, Figure 4C). We also checked the mutation status of *PDLIM2* in 94 ESCC cases with mutation data available (Figure S2). Among 94 ESCC patients, two had missense mutations (Figure S2A). However, no survival difference was observed between the mutation and no mutation group (Figure S2B).

**Figure 5:**
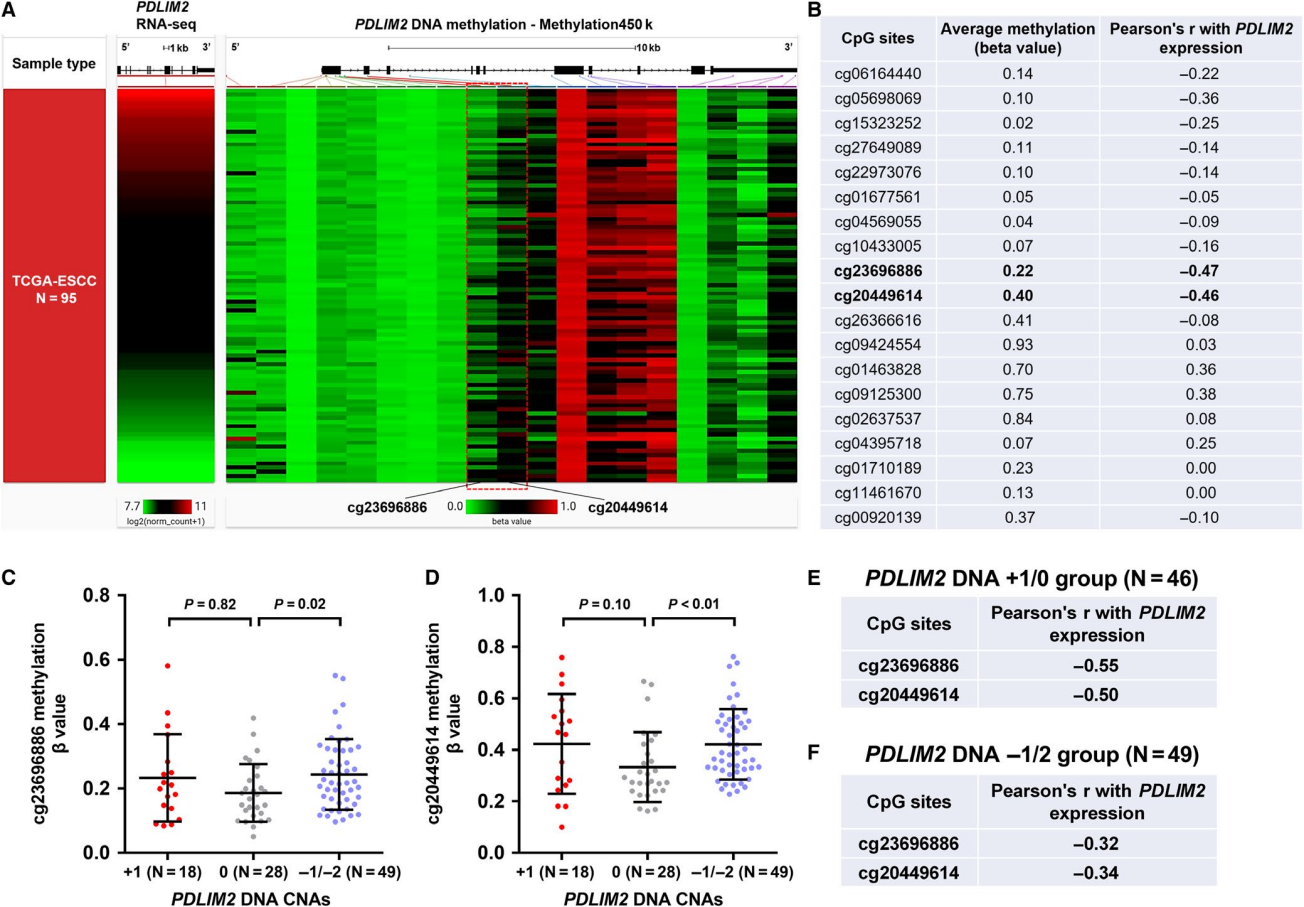
*PDLIM2* DNA methylation and expression. (A,B), Heatmap (A) and statistical summary of the Pearson's correlation coefficient (B) between the methylation level (beta value) of CpG sites in *PDLIM2* DNA locus and *PDLIM2* expression. (C,D), Plot charts comparing the methylation level of cg23696886 (C) and cg20449614 (D) in different *PDLIM2* CNA groups. (E,F), the Pearson's correlation coefficient between the methylation level of the two sites and *PDLIM2* expression in copy neutral/amplification (0/+1) (E) group and copy loss group (−1/−2) (F)

We then checked methylation 450 k data of 95 ESCC cases in TCGA. The methylation status of 19 CpG sites in *PDLIM2* DNA locus was measured (Figure 5A). The correlation between the methylation level of *PDLIM2* CpG sites and total *PDLIM2* expression was calculated (Figure 5B). Results showed that the methylation level of two CpG site (cg23696886 and cg20449614) had a moderately negative correlation with *PDLIM2* expression (Pearson's *r* = −0.47 and *r* = −0.46 respectively; Figure 5B). cg23696886 is located at the intron before exon 2, while cg20449614 is in exon 2 (red‐dotted frame, Figure 5A), where the *PDLIM2* promoter maps to Ref.^19^.

Then, we analysed the correlation between *PDLIM2* CNAs and the methylation of the two CpG sites. Results showed that the copy loss group (−1/−2) had significantly higher methylation at these two CpG sites (*P* = 0.02 and *P* = < 0.01 respectively, Figure 5C,D). To reduce the potential mixing effect of CNAs and methylation on gene expression, we analysed the correlation between *PDLIM2* expression and the methylation of the two CpG sites in copy neutral/amplification group and copy loss group respectively (Figure 5E,F). Results showed that, in copy neutral/amplification group, there were still moderately negative correlations (Figure 5E). However, the correlation coefficients were much smaller in the DNA −1/−2 groups (Figure 5F). These findings imply that DNA hypermethylation might be a potential mechanism leading to *PDLIM2* down‐regulation in copy neutral/amplification group. In comparison, *PDLIM2* copy loss might be the dominant cause of reduced gene expression in the copy loss group.

## 4 DISCUSSION

In this study, by combined use of the microarray data in GDS3838 and TCGA‐ESCC, we found that *PDLIM2* was the only candidate gene (out of over 15,000 screened genes) that had at least fourfold change and was associated with nodal invasion and survival outcome at the same time. Besides, univariate and multivariate analysis confirmed that *PDLIM2* expression was independently associated with longer OS in ESCC patients (HR: 0.64, 95% CI: 0.43‐0.95, *P* = 0.03), after adjustment for gender and pathologic stages. These findings suggest that *PDLIM2* expression might have potential prognostic value in ESCC. To the best of our knowledge, this is the first report showing the down‐regulation of *PDLIM2* in ESCC had independent prognostic value.

*PDLIM2* gene maps to 8p21 in human genome and encoded a protein called Pdz And Lim Domain Protein 2 (PDLIM2), which is also known as Mystique or SLIM. PDLIM2 belongs to the actinin‐associated LIM family of proteins and participate in the cellular processes such as cell differentiation and cytoskeleton organization as it interacts with α‐actinin‐1, α‐actinin‐4, filamin A and myosin heavy polypeptide 9 in epithelial cells.^19^, ^20^ In addition, it acts as ubiquitin E3‐ligase that induces the inactivation and degradation of STAT1, STAT3 and STAT4, thereby negatively regulates STAT signalling pathway hemopoietic cells.^21^, ^22^ Previous studies found that *PDLIM2* had both tumour suppressive and oncogenic effects in different malignancies. Its up‐regulation was observed in androgen‐independent prostate cancer cell lines.^23^ Inhibition of its expression could induce G2/M cell cycle arrest and reduce the malignant phenotypes of the cancer cells, such as proliferation, clonogenicity and invasiveness.^23^ In Merlin‐deficient Meningioma and Schwannoma, *PDLIM2* up‐regulation is associated with increased proliferation of tumour cells.^24^ In comparison, *PDLIM2* expression was down‐regulated in some other tumours, such as colorectal cancer,^25^, ^26^ breast cancer,^27^ ovarian cancer,^28^ classical Hodgkin and anaplastic large cell lymphoma.^29^ It acts as an essential terminator of NF‐κB activation in both colorectal cancer and breast cancer cells.^26^, ^27^ Its expression results in retarded anchorage‐independent growth in vitro and decreased tumourigenicities of the cancer cells in vivo.^26^, ^27^ The Luminal A breast cancer patients who had preserved *PDLIM2* expression also had longer relapse‐free survival.^30^ *PDLIM2* can suppress ovarian cancer growth, by decreasing endogenous NO level and subsequent M2 type tumour‐associated macrophage infiltration.^28^ These findings suggest that the expression and functional role of *PDLIM2* might be cancer specific.

For PDZ containing proteins, alternative splicing is an important source of functional diversity.^31^ For *PDLIM2*, alternative splicing and subsequent transcript variants encoding multiple isoforms have been observed.^19^ This might help to explain the functional difference of this gene in different cancers. The diversity of the transcripts might also impair the prognostic value of this gene if we only consider its total expression. Therefore, we decided to explore the prognostic value of individual exons of this gene. By checking *PDLIM2* transcript isoforms in TCGA‐ESCA, we found that the canonical uc003xby.4 is the dominant transcript, which is formed by 10 exons. Subsequent analysis confirmed that exon 7/8/9/10 expression of this transcript also independently predicted favourable OS of ESCC patients and might be a better prognostic marker than total *PDLIM2* expression.

The segment where exon 7/8/9/10 maps is a critical area of alternative splicing. Exon 9/10 is essential for the integrity of the LIM domain of PDLIM2. The absence of exon 9 results in premature termination and is predicted to encode a PDZ‐only protein 19. The LIM domain of PDLIM2 directly interacts with some important signalling molecules, including kinases, receptors, and phosphatases.^19^, ^20^ It also mediates the ubiquitination of nuclear p65.^32^ In addition, this domain is involved in regulating the cellular distribution of PDLIM2.^33^ Deletion of this domain results in increased accumulation of PDLIM2 in the cytoplasm and nucleoplasm, and reduced protein level in the nuclear matrix.^33^ These findings suggest that the expression of exon 1‐10 might be critical to maintaining the normal function of PDLIM2 in ESCC.

Copy number alterations are common genetic alterations that contribute to gene dysregulation in ESCC.^34^, ^35^ Some of the previously observed CNAs include amplification of 11q13.3 (*FGF4*), 3p26.33 (*SOX2OT*), 8q24.21 (*MYC*), 14q21.1 (*FOXA1*) and deletion of 9p21.3 (*CDKN2A*).^35^ Some tumour suppressor genes in 8p21 were down‐regulated as a result of deleted chromosomal region, such as *BNIP3L* in breast and ovarian cancer^36^ and *NKX3.1* and miR‐3622b in prostate cancer.^37^, ^38^ This triggered our interest to explore the association between the CNA status of *PDLIM2* and its expression in ESCC. Using data in TCGA‐ESCC, we found that *PDLIM2* gene‐level deletion is frequent and is associated with decreased gene expression, suggesting that DNA copy loss might be a mechanism of its down‐regulation. Some previous studies reported that *PDLIM2* expression was also regulated by the promoter methylation that blocks its transcription in colon cancer,^26^ ovarian cancer^28^ and classical Hodgkin and anaplastic large cell lymphoma.^29^ However, whether its expression is related to the methylation status of its promoter in ESCC is still not clear. In this study, we found the methylation status of two CpG sites (cg23696886 and cg20449614) in the proximal promoter region of *PDLIM2* showed a moderately negative correlation with the gene expression, especially in *PDLIM2* copy neutral/amplification group. Therefore, we infer that promoter hypermethylation might also be an important epigenetic alteration leading to suppressed *PDLIM2* expression in ESCC.

This study also has some limitations. This is an in silico analysis based on a relatively small sample size. Additional validation with a bigger dataset could be considered in the future. Besides, the functional role of *PDLIM2* in ESCC was not explored in the current study. Molecular studies are required to explore the potential suppressive effect of PDLIM2 on the malignant phenotype of ESCC and the underlying mechanisms.

## 5 CONCLUSION

In conclusion, by the systemic screening in this study, we found that *PDLIM2* is one of the most significant down‐regulated genes in ESCC and might be a novel prognostic predictor in terms of OS. The expression of *PDLIM2* exon 7/8/9/10 had the best prognostic value. *PDLIM2* down‐regulation is associated with gene‐level copy deletion and promoter hypermethylation.

## CONFLICT OF INTEREST

The authors confirm that there are no conflict of interest.

## AUTHORS CONTRIBUTIONS

Conceptualization, Guiqin Song and Jun Xu; Methodology, Guiqin Song, Lang He and Xiao Sun; Software, Guiqin Song, Rong Xiong and Xin Hu; Validation, Ruolan Zhang and Qiuju Yue; Formal Analysis, Guiqin Song, Jun Xu, Lang He and Yuxi Luo; Data Curation, Kang Liu and Gang Feng; Writing – Original Draft Preparation, Guiqin Song and Jun Xu; Writing – Review and Editing, Kang Liu and Gang Feng; Supervision, Kang Liu and Gang Feng; Project Administration, Kang Liu and Gang Feng.

## Data Availability

All data used in this study were included in the manuscript and supplementary materials.
