# Supplementary material for: Systematic profiling identifies PDLIM2 as a novel prognostic predictor for oesophageal squamous cell carcinoma (ESCC)
Source: J Cell Mol Med. 2019 Jun 20;23(8):5751–61. doi: 10.1111/jcmm.14491 (PMC6653303; doi:10.1111/jcmm.14491)
Supplement: Supplementary file 1 [file JCMM-23-5751-s001.docx]

**Supplementary figure 1. ROC curves and the AUCs of the 18 OS related genes**


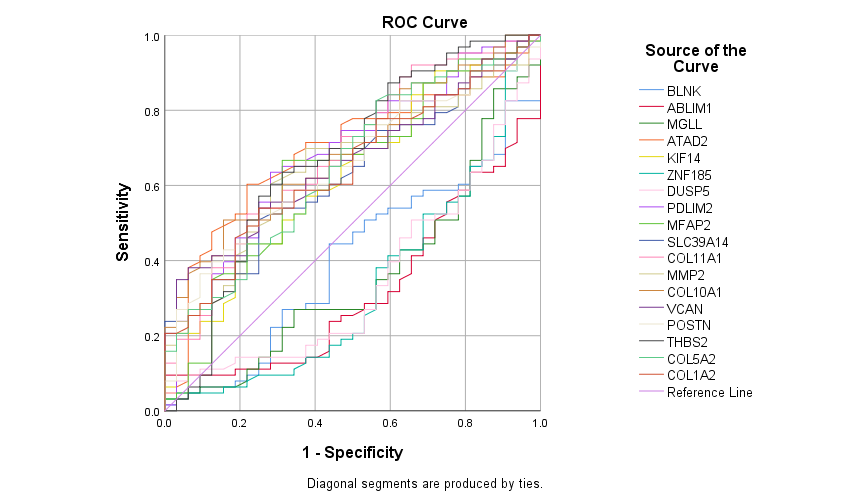


| **Area Under the Curve** | | | | | |
| --- | --- | --- | --- | --- | --- |
| Test Result Variable(s) | Area | Std. Error^a^ | Asymptotic Sig.^b^ | Asymptotic 95% Confidence Interval | |
|  |  |  |  | Lower Bound | Upper Bound |
| BLNK | .400 | .060 | .113 | .283 | .518 |
| ABLIM1 | .322 | .056 | .005 | .212 | .432 |
| MGLL | .358 | .060 | .025 | .241 | .476 |
| ATAD2 | **.692** | .056 | .002 | .583 | .802 |
| KIF14 | .626 | .060 | .045 | .509 | .743 |
| ZNF185 | .335 | .061 | .009 | .215 | .454 |
| DUSP5 | .350 | .059 | .018 | .234 | .467 |
| PDLIM2 | **.684** | .057 | .004 | .572 | .796 |
| MFAP2 | .639 | .061 | .027 | .520 | .759 |
| SLC39A14 | .631 | .057 | .038 | .518 | .743 |
| COL11A1 | .683 | .057 | .004 | .571 | .796 |
| MMP2 | .656 | .057 | .013 | .545 | .767 |
| COL10A1 | **.691** | .054 | .002 | .584 | .797 |
| VCAN | .661 | .055 | .010 | .553 | .770 |
| POSTN | .654 | .057 | .014 | .543 | .765 |
| THBS2 | .674 | .062 | .006 | .552 | .795 |
| COL5A2 | .652 | .059 | .016 | .537 | .766 |
| COL1A2 | .648 | .057 | .019 | .536 | .759 |
| The test result variable(s): ABLIM1, MGLL, ATAD2, KIF14, ZNF185, DUSP5, PDLIM2, MFAP2, SLC39A14, MMP2, COL10A1, VCAN, POSTN, THBS2, COL5A2, COL1A2 has at least one tie between the positive actual state group and the negative actual state group. Statistics may be biased. | | | | | |
| a. Under the nonparametric assumption | | | | | |
| b. Null hypothesis: true area = 0.5 | | | | | |
