# Supplementary figures and images for: Systematic profiling identifies PDLIM2 as a novel prognostic predictor for oesophageal squamous cell carcinoma (ESCC)
Source: J Cell Mol Med. 2019 Jun 20;23(8):5751–61. doi: 10.1111/jcmm.14491 (PMC6653303; doi:10.1111/jcmm.14491)

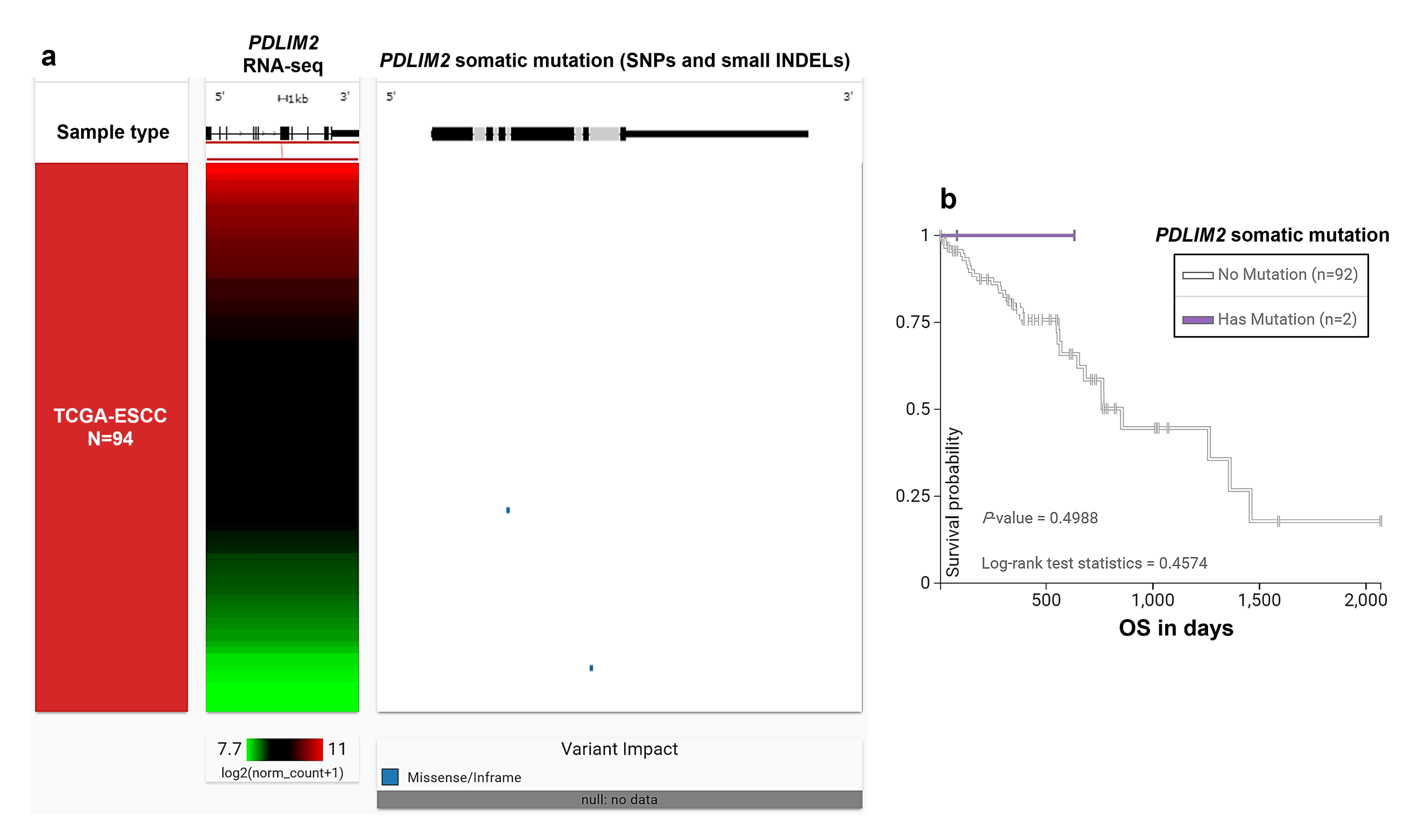

Supplement: Supplementary file 2 [file JCMM-23-5751-s002.tif]
