# Supplementary material for: Systematic profiling identifies PDLIM2 as a novel prognostic predictor for oesophageal squamous cell carcinoma (ESCC)
Source: J Cell Mol Med. 2019 Jun 20;23(8):5751–61. doi: 10.1111/jcmm.14491 (PMC6653303; doi:10.1111/jcmm.14491)
Supplement: Supplementary file 5 [file JCMM-23-5751-s005.docx]

**Supplementary table 3. Stepwise regression analysis of the value of total *PDLIM2* and its exon 7/8/9/10 expression as predictive variables for OS in ESCC patients**

| **Coefficients^a^** | | | | | | |
| --- | --- | --- | --- | --- | --- | --- |
| Model | | Unstandardized Coefficients | | Standardized Coefficients | t | Sig. |
|  |  | B | Std. Error | Beta |  |  |
| 1 | (Constant) | .880 | .161 |  | 5.455 | .000 |
|  | chr8:22446663-22451810:+ | -.256 | .073 | -.342 | -3.512 | .001 |
| a. Dependent Variable: OS | | | | | | |

| **Excluded Variables^a^** | | | | | | |
| --- | --- | --- | --- | --- | --- | --- |
| Model | | Beta In | t | Sig. | Partial Correlation | Collinearity Statistics |
|  |  |  |  |  |  | Tolerance |
| 1 | PDLIM2 | 1.023^b^ | 2.464 | .016 | .249 | .052 |
| a. Dependent Variable: OS | | | | | | |
| b. Predictors in the Model: (Constant), chr8:22446663-22451810:+ | | | | | | |

Both *PDLIM2* and the segment indicating the expression of exon 7/8/9/10 were put in the model for analysis. Model 1 shows the best model with the best predictor. *PDLIM2* expression was the excluded variable.
